# 1436. Interventions to Address the Global Underreporting of Pediatric and Adolescent Tuberculosis: A Systematic Review

**DOI:** 10.1093/ofid/ofac492.1265

**Published:** 2022-12-15

**Authors:** Alexandra R Linn, Andrew Steenhoff

**Affiliations:** Hospital of the University of Pennsylvania/Children's Hospital of Philadelphia, Philadelphia, Pennsylvania; Children's Hospital of Philadelphia, Philadelphia, Pennsylvania

## Abstract

**Background:**

The WHO currently estimates that at least a third of pediatric and adolescent tuberculosis cases go untreated or incompletely treated each year. Many cases are detected but lost to follow-up due to underreporting to national or private surveillance systems.

**Methods:**

We conducted a systematic literature review to better understand the global reporting gap of pediatric tuberculosis as well as current interventions. Following PRISMA guidelines, the review included articles published in English within the past 30 years. Studies were included if they discussed underreporting of tuberculosis in pediatric and adolescent patients. Studies were excluded if they did not include these populations or if they focused on challenges with initial diagnosis or general loss to follow-up rather than underreporting. In May and June 2021, we searched PubMed and Ovid databases along with reference lists of included works. Multiple reviewers and further assessments of heterogeneity and bias will be implemented in the next steps of the project.

**Results:**

Of 458 records identified, 420 were screened for inclusion and 19 studies met inclusion criteria in the preliminary search. Of included studies, 14 described the gap in reporting of pediatric tuberculosis and 5 described potential interventions to close this gap. The estimated gap in reporting ranged from 16% to 98% and was described in 23 countries. Larger gaps were described in rural areas, in areas with higher disease burden, in patients with disseminated disease, and in those under age 5 years. Effective interventions are limited and include strengthening linkage systems between hospitals and clinics, increasing communication between patients and healthcare workers following initial diagnosis, utilizing electronic systems to optimize ongoing monitoring, improving death registration, and using implementation science for continued quality improvement.

**Table 1:**
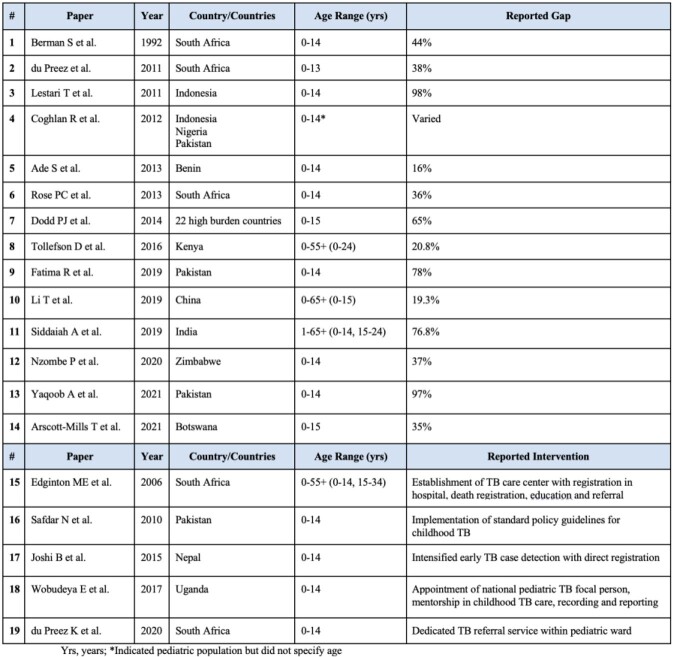

Characteristics of studies included in the review

**Conclusion:**

Underreporting of pediatric and adolescent tuberculosis is a significant global problem. Few evidence-based effective strategies exist to close this gap. Future research should assess the ability of creative, pragmatic solutions to mitigate this with an emphasis on pediatric populations in high-TB burden countries.

**Disclosures:**

**All Authors**: No reported disclosures.

